# A novel protein FNDC3B-267aa encoded by circ0003692 inhibits gastric cancer metastasis via promoting proteasomal degradation of c-Myc

**DOI:** 10.1186/s12967-024-05225-4

**Published:** 2024-05-27

**Authors:** Yu-Ying Liu, Yu-Ying Zhang, Ling-Yu Ran, Bo Huang, Jun-Wu Ren, Qiang Ma, Xiao-Juan Pan, Fei-Fei Yang, Ce Liang, Xiao-Lin Wang, Shi-Min Wang, Ai Ran, Hao Ning, Yan Jiang, Chang-Hong Qin, Bin Xiao

**Affiliations:** 1 College of Pharmacy, Chongqing Medical University, Chongqing, 400016 P.R. China; 2 Department of Kidney, Southwest Hospital, Army Medical University, Chongqing, 400038 P.R. China

**Keywords:** circ0003692, FNDC3B-267aa, c-Myc, Proteasomal degradation, Gastric cancer

## Abstract

**Background:**

Gastric cancer (GC) ranks fifth in global cancer incidence and third in mortality rate among all cancer types. Circular RNAs (circRNAs) have been extensively demonstrated to regulate multiple malignant biological behaviors in GC. Emerging evidence suggests that several circRNAs derived from FNDC3B play pivotal roles in cancer. However, the role of circFNDC3B in GC remains elusive.

**Methods:**

We initially screened circFNDC3B with translation potential via bioinformatics algorithm prediction. Subsequently, Sanger sequencing, qRT-PCR, RNase R, RNA-FISH and nuclear-cytoplasmic fractionation assays were explored to assess the identification and localization of circ0003692, a circRNA derived from FNDC3B. qRT-PCR and ISH were performed to quantify expression of circ0003692 in human GC tissues and adjacent normal tissues. The protein-encoding ability of circ0003692 was investigated through dual-luciferase reporter assay and LC/MS. The biological behavior of circ0003692 in GC was confirmed via in vivo and in vitro experiments. Additionally, Co-IP and rescue experiments were performed to elucidate the interaction between the encoded protein and c-Myc.

**Results:**

We found that circ0003692 was significantly downregulated in GC tissues. Circ0003692 had the potential to encode a novel protein FNDC3B-267aa, which was downregulated in GC cells. We verified that FNDC3B-267aa, rather than circ0003692, inhibited GC migration in vitro and in vivo. Mechanistically, FNDC3B-267aa directly interacted with c-Myc and promoted proteasomal degradation of c-Myc, resulting in the downregulation of c-Myc-Snail/Slug axis.

**Conclusions:**

Our study revealed that the novel protein FNDC3B-267aa encoded by circ0003692 suppressed GC metastasis through binding to c-Myc and enhancing proteasome-mediated degradation of c-Myc. The study offers the potential applications of circ0003692 or FNDC3B-267aa as therapeutic targets for GC.

**Graphical abstract:**

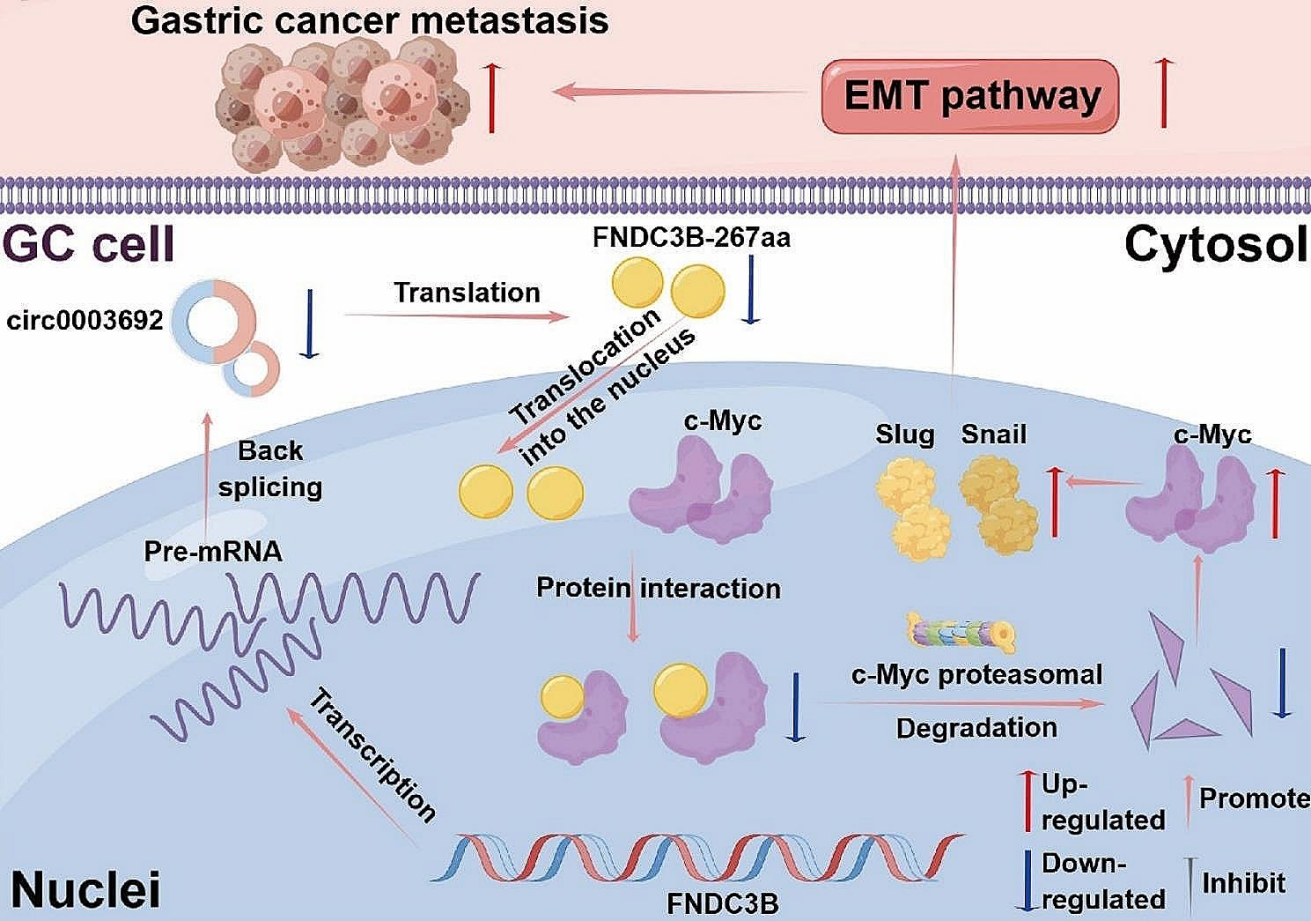

The mechanism of circ0003692 in suppressing metastasis of GC. FNDC3B-267aa encoded by circ0003692 interacted with c-Myc and promoted the proteasomal degradation of c-Myc, thereby down-regulated c-Myc-Snail/Slug axis and EMT pathway.

**Supplementary Information:**

The online version contains supplementary material available at 10.1186/s12967-024-05225-4.

## Highlights

Circ0003692 is decreased in GC and associated with T, N, M grade.

Circ0003692 encodes a novel protein FNDC3B-267aa.

Circ0003692 inhibits GC migration by translating FNDC3B-267aa.

FNDC3B-267aa interacts with c-Myc and promotes its proteasomal degradation.

**Supplementary Information:**

The online version contains supplementary material available at 10.1186/s12967-024-05225-4.

## Introduction

Gastric cancer (GC) ranks fifth in global cancer incidence and third in cancer-related mortality [1, 2]. GC is characterized as an invasive and heterogeneous malignant tumor [1, 3], with a median overall survival of merely 16 months [1, 3, 4]. Tumor metastasis accounts to 90% of cancer-related death [5]. Patients with late-stage typically exhibit signs of infiltration and metastasis, especially liver metastasis [6]. However, effective treatment for metastatic GC remains elusive [7].

CircRNA forms a closed-loop structure, rendering it more stable and conserved [8]. Recently, there is compelling evidence indicating that circRNA plays a vital role in GC proliferation, invasion, migration, and apoptosis [9–11], suggesting its potential as a diagnostic marker and therapeutic target for GC [10]. CircFNDC3B, derived from the gene FNDC3B, stands as one of the extensively studied circRNAs. Fourteen studies have investigated the diverse functions of circFNDC3B in various diseases [12]. For example, Hong et al. demonstrated that circFNDC3B promoted the migration and invasion of GC cells by regulating the expression of E-cadherin and CD44 [13]. However, the role of circFNDC3B in the progression of GC remains elusive.

Currently, the functional mechanisms of circRNA encompass several aspects, including acting as a miRNA sponge [11, 12], binding to RNA binding proteins (RBPs) [14] or translating protein [15]. Recently, there has been significant emphasis on translational circRNA to determine its clinical significance [16]. However, it is unclear whether circFNDC3B can encode a protein and regulate progression of GC. In this study, we predict circFNDC3B with translational potential in GC. Our investigation revealed that circ0003692 exhibited low expression level in GC and had the capability to encode a novel protein consisting of 267 amino acids, denoted as FNDC3B-267aa. In vitro and in vivo experiments provided evidences that FNDC3B-267aa diminished the migratory capacity of GC. Furthermore, our finding indicated that FNDC3B-267aa inhibited the c-Myc-Snail/Slug axis by promoting the proteasome-mediated degradation of c-Myc.

## Materials and methods

### Patients and tissue samples

This study comprised 64 clinical cases of GC patients who were diagnosed with GC by histopathological assessment and underwent curative resection. All tissues were obtained from Southwest Hospital Affiliated with Army Medical University (Additional file 1: Table S1). Fresh specimens of GC and adjacent normal tissue (NC) samples were surgically resected. All specimens required for the experiment were approved by the Ethics Committee of the Southwest Hospital Affiliated to the Army Medical University. After three-dimensional storage, the specimens were placed in RNAlater (ThermoFisher, Shanghai, China) at 4 ℃, and then transferred to a refrigerator at -80 ℃.

### Plasmids, siRNAs and cell transfection

The circ0003692 siRNA (si-circ0003692) and NC siRNA used in the experiment were synthesized by RiboBio (Guangzhou, China). The FNDC3B siRNA (si-FNDC3B) and c-Myc siRNA (si-c-Myc) used in the experiment were synthesized by Genepharma (Shanghai, China) and Tsingke Biotech (Jiangsu, China), respectively. IRES1, IRES2, IRES-FL and circAKT-IRES were synthesized and cloned into Luc2-IRES-Report vector using the KpnI and EcoRI sites. Empty vector and Luc2-ECMV-IRES-Report were purchased from GENESEED (Guangzhou, China). The full length of circ0003692 marked with 3×flag was synthesized and cloned into pLC5-ciR vector using the BamHI and EcoRI sites. The linear DNA sequence of FNDC3B-267aa was synthesized and cloned into vector pCDH using the EcoRI and BgLII sites. The c-Myc overexpressed vector were purchased from Leqin Biotech (Chongqing, China). The control plasmid (pLC5-IRES-GFP) and pCDH were purchased from Tsingke (Jiangsu, China). We conducted plasmid transfections using NEOFECT™ DNA transfection reagent (Neofect Biotech, Beijing, China) in adherence to the manufacturer’s provided guidelines. We conducted plasmid transfections using Lipo2000™ Transfection Reagent (Invitrogen, USA) in adherence to the manufacturer’s provided guidelines. All siRNA sequences are listed in Additional file 1: Table S2.

### Tissue microarray (TMA) and in situ hybridization (ISH) experiment

TMA samples, sourced from Outdo Biotech (Shanghai, China), were derived from 180 GCs tissues and adjacent normal tissues (Additional file 1: Table S3). The clinical data and demographic were publicly available and provided by Aoduo Biology (Shanghai, China). The expression of circ0003692 in GC tissue was assessedby ISH, following the manufacturer’s guidelines. The specific digoxin-labeled circ0003692 probe and the ISH kit were procured from Boster (Wuhan, China). According to our previous study, the expression of circ0003692 was quantified and analyzed to calculate the ISH staining score [17]. The probe sequence was provided in Additional file 1: Table 4.

### Cycloheximide and proteasome inhibitor rescue assays

AGS and HGC-27 were transfected with pCDH-FNDC3B-267aa for 48 h. To verify target protein stability, we employed eukaryotic protein synthesis inhibitor cycloheximide (CHX) (Genview, Beijing, China) at a concentration of 100 µM for varying durations of 0, 30, 90, and 180 min. Furthermore, MG132 (MedChemExpress, New Jersey, USA), recognized for its ability to inhibit 26 S proteasome complexes that target ubiquitin-binding proteins, was utilized. The transfected cells were subjected to treatment with MG132 (10 µM) for the same time intervals (0, 30, 90, and 180 min).

### Migration assay

Wound healing and transwell migration assays were conducted to assess the migration ability of GC cells. Generally, the upper chamber is filled with 200 µL serum-free media containing 5 × 10^4^ cells, while the lower 24-well plate is filled with 650 µL serum-containing media. After incubation for 20 h, transwell chambers were fixed with 4% paraformaldehyde for 15 min, and then stainedwith 1% crystal violet for 15 min. Images of the transwell chambers were captured and quantified using ImageJ software. The GC cells transfected in six-well plate were scratched 48 h after transfection. The wound width from random fields was measured at 0, 12 and 24 h. Wound width were conducted using ImageJ.

### RNA-fluorescence in situ hybridization (RNA-FISH)

GC cells were cultured on an 8-well chamber slide obtained from Ibidi (Martinsried, Germany) for 24 h. Subsequently, a commercial reagent kit (GenePharma, Shanghai, China) was employed for FISH analysis according to the manufacturer’s guidelines. The biotin-labeled probe was synthesized by Genepharma (Additional file 1: Table S5).

### Immunofluorescence (IF) assay

The IF assay was performed to determine the subcellular localization of FNDC3B-267aa and c-Myc, as described previously. Cells were seeded in a confocal 8-well plate with 4% paraformaldehyde for 15 min, followed by permeabilization with 0.5% TritonX-100 in PBST for 15 min. Blocking was achieved with 5% BSA. After 1 h, primary antibodies were then applied and incubated with the cells overnight at 4 °C. The second day, PBST dishes were used for three times, and the secondary antibody conjugated with fluorescent dye was incubated for 1 h. Finally, DAPI was added to avoid light and incubated for 5 min. Fluorescence images were acquired using a microscope and analyzed with manufacturer-provided software.

### Immunohistochemistry (IHC) assay

IHC analysis was conducted on mouse xenograft tumor tissue following standard protocols. Subsequently, the tumor tissue sections were sent to Biossci (Wuhan, China) for immunohistochemical staining.

### Immunoprecipitation-mass spectrometry (LC-MS) analysis

LC-MS analysis was conducted by technology company (Sangon Biotech, Shanghai, China). The protein underwent separation via SDS-PAGE and overnight digestion with trypsin hydrolysis. Subsequently, the peptide solution resulting from enzymatic hydrolysis was desalted using a desalting column. The digested peptides were analyzed with a Q Exactive Plus mass spectrometer (ThermoFisher Scientific, USA). The fragment spectra were analyzed using the ProteinPilot (V4.5).

### Quantitative real‑time PCR assay (qRT-PCR)

Total cellular RNA was extracted by Trizol (Takara, Japan). Reverse transcription was performed using the PrimeScript™ RT reagent Kit (Takara, Japan), followed by qPCR analysis of RNA expression levels using 2×SP qPCR Mix (Bioground, China). GAPDH was utilized as a normalization control. Divergent and convergent primers of circ0003692 and GAPDH were employed to assess the circular characteristics of circ0003692 by qRT-PCR. All primers in this study were synthesized by Tsingke Biotechnology (Beijing, China). Primer sequences were listed in Additional file 1: Table S6.

### Subcellular fractionation assay

The nucleoplasmic separation kit (Invitrogen) was used for subcellular fractionation assay referring to the manufacturer’s guidelines. All primer sequences in this study are listed in Table S5 (Additional file 1).

### In vivo liver metastasis assay

Nude mice obtained from the Shanghai Model Biology Center were randomly grouped based on their body weight. Subsequently, tably transfected AGS cells (10^6^ cells/0.1 mL PBS; *n* = 3 per group) were injected into the spleens of the nude mice. After 60 days, the mice were euthanized, and liver tissues were harvested for fixation and immunohistochemical staining. All animal experiments were conducted in accordance with the protocol approved by the Institutional Animal Care and Use Committee of Chongqing Medical University, Chongqing.

### Statistical analysis

Statistical differences were calculated by GraphPad Prism 7.0 and SPSS 18.0, then presented as mean ± standard deviation (SD). Differences between two groups were analyzed by Chisquare test and Student’s t-test. One-way ANOVA was employed to compare continuous variables among multiple groups. ISH and IHC score were analysed by the Mann–Whitney U test. Statistical significance was set at *P* < 0.05.

## Results

### Identification of downregulated hsa_circ_0003692 with translation potential in GC tissues and cells

**Fig. 1:**
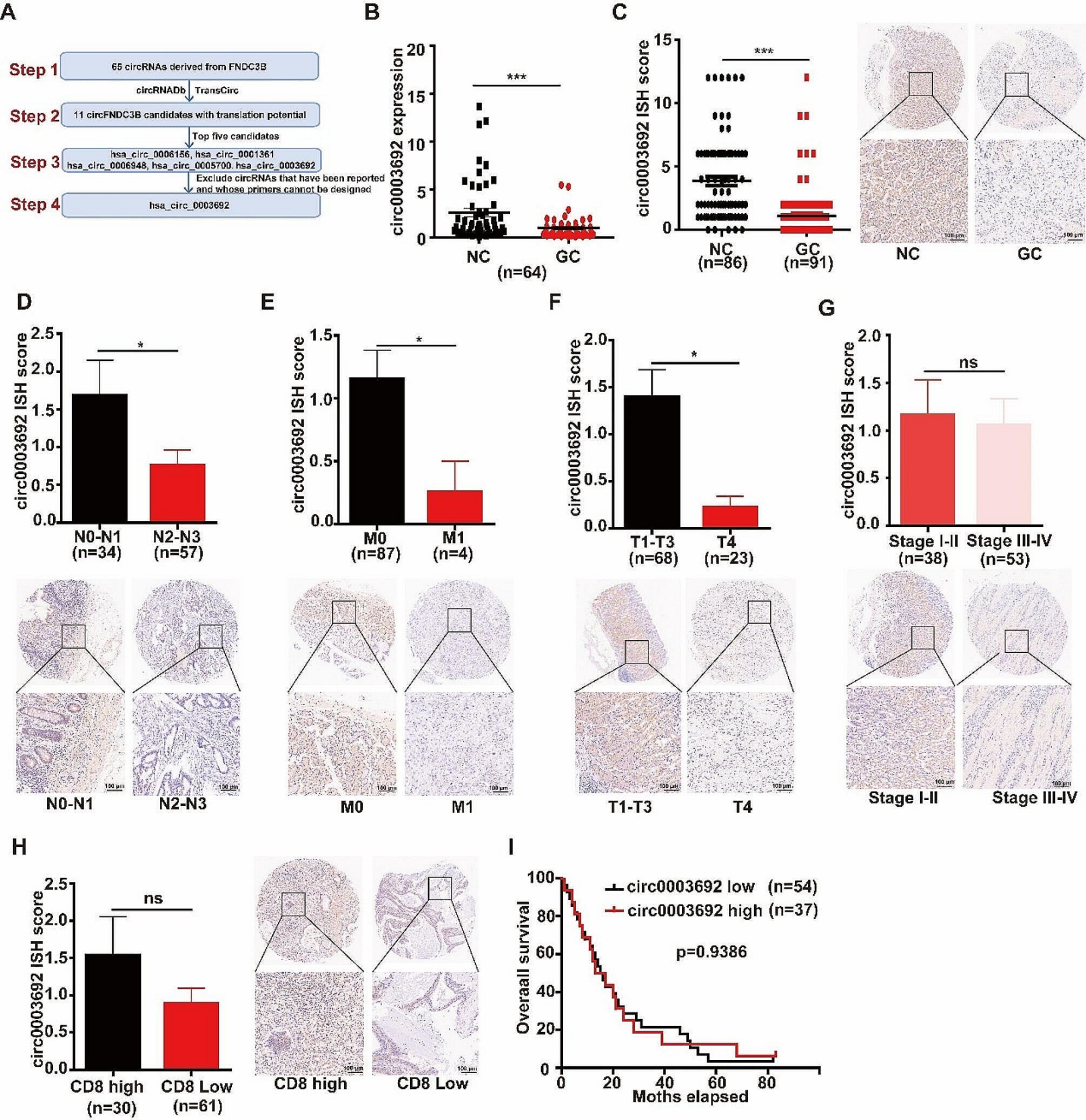
Identification of downregulated circ0003692 with translation potential in GC tissues and cells. **(A)** The screening strategy of circRNAs derived from FNDC3B and with translation potential in GC. **(B)** Assessment of circ0003692 expression in GC tissues and corresponding adjacent tissues (*n* = 64). **(C)** ISH examination of circ0003692 expression in TMA from GC patients (adjacent tissues = 86 cases; GC tissues = 94 cases). **D-H.** ISH analysis of circ0003692 expression and representative images of circ0003692 staining in GC patients with various TNM grade, stage, and CD8^+^ staining. **I.** Survival analysis of GC patients according to low and high levels of circ0003692 expression. *P < 0.05, ***P < 0.001, ns, no significance

We ultimately identified 11 circFNDC3B candidates that capable of translating proteins among 65 circFNDC3Bs via circRNADb and TransCirc database. Among the top five candidates, hsa_circ_0001361 and hsa_circ_0006156 have been reported in oral cancer and colon cancer [12], and the primers for hsa_circ_0006948 and hsa_circ_0005700 were not successfully designed. Therefore, we selected circ0003692 for intensive study (Additional file 1: Table S7, Fig. 1A).

Next, we assessed the expression of circ0003692 in 64 GC tissues and their corresponding non-cancerous tissues using qRT-PCR, revealing a significant decrease in circ0003692 expression in the cancerous tissues (Fig. 1B). Subsequently, we utilized a tissue microarray consisting of 94 GC tissues and 86 adjacent tumor tissues for ISH to further investigate the clinical relationship of circ0003692. The results indicated that the ISH score of circ0003692 in GC tissues was lower compared to that in corresponding NC tissues (Fig. 1C). Moreover, the expression of circ0003692 was reduced in GC tissues with advanced T grade, higher N grade, or M grade (Fig. 1D-F). Furthermore, patients at more advanced stage exhibited lower circ0003692 expression, although statistical significance was not reached (Fig. 1G). Interestingly, circ0003692 expression was lower in GC tissues displayed less CD8^+^ expression (Fig. 1H). However, the expression of circ0003692 did not correlate with the overall survival of GC patients (Fig. 1I). In summary, our findings indicated that circ0003692 was significantly underexpressed in GC, suggesting its potential anti-tumor role in GC.

### Characterization of circ0003692 in GC

**Fig. 2:**
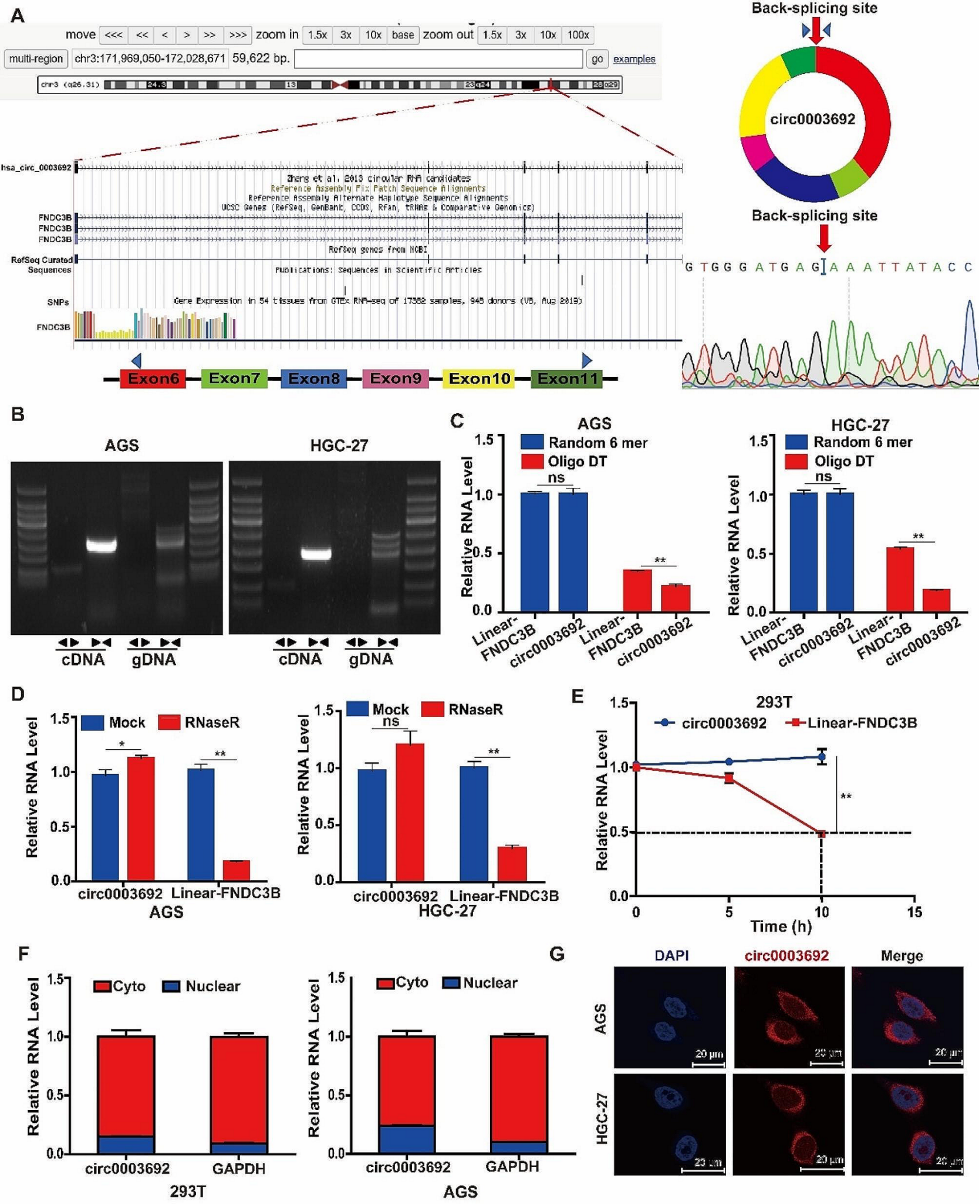
Characteristics of circ0003692. **A**. Genomic structure and formation of circ0003692 was shown. The specific divergent and convergent primers were designed, as depicted. The back-splice junction site of circ0003692 was verified through sanger sequencing. **(B)** Amplification of circ0003692 using divergent primers from cDNA but not gDNA. **(C)** Expression of circ0003692 and FNDC3B amplified from total RNA of AGS and HGC-27 cells reverse transcribed by random 6 mers or oligo dT primers. **(D)** Effect of RNase R treatment on circ0003692 and Linear FNDC3B mRNA expression. **(E)** Effect of Actinomycin D treatment on circ0003692 and linear FNDC3B mRNA expression. **(F)** qRT-PCR for the distribution of circ0003692, FNDC3B and GAPDH in the cytoplasmic and nuclear fractions. **(G)** Localization of circ0003692 in 293T and AGS cells using RNA-FISH.*P < 0.05, **P < 0.01, ns, no significance

Based on circBase, circ0003692 originates from exon 6 to 11 of the FNDC3B gene, located on chr3 (Fig. 2A). Our findings confirmed the exclusive amplification of circ0003692 from cDNA using divergent primers, whereas linear-FNDC3B mRNA was amplified from both cDNA and gDNA templates using convergent primers (Fig. 2B). The utilization of random primers, instead of oligo dT primers, for the reverse transcription of circ0003692 verified its circular structure (Fig. 2C). Additionally, upon treatment with RNaseR or ActinomycinD, circ0003692 exhibited greater stability compared to linear-FNDC3B (Fig. 2D-E). Subcellular distribution analysis and FISH further revealed that circ0003692 localized in the cytoplasm (Fig. 2F-G). In summary, these results demonstrated the robust stability of circ0003692 as a circRNA in GC cells.

### Circ0003692 encodes a novel protein FNDC3B-267aa

**Fig. 3:**
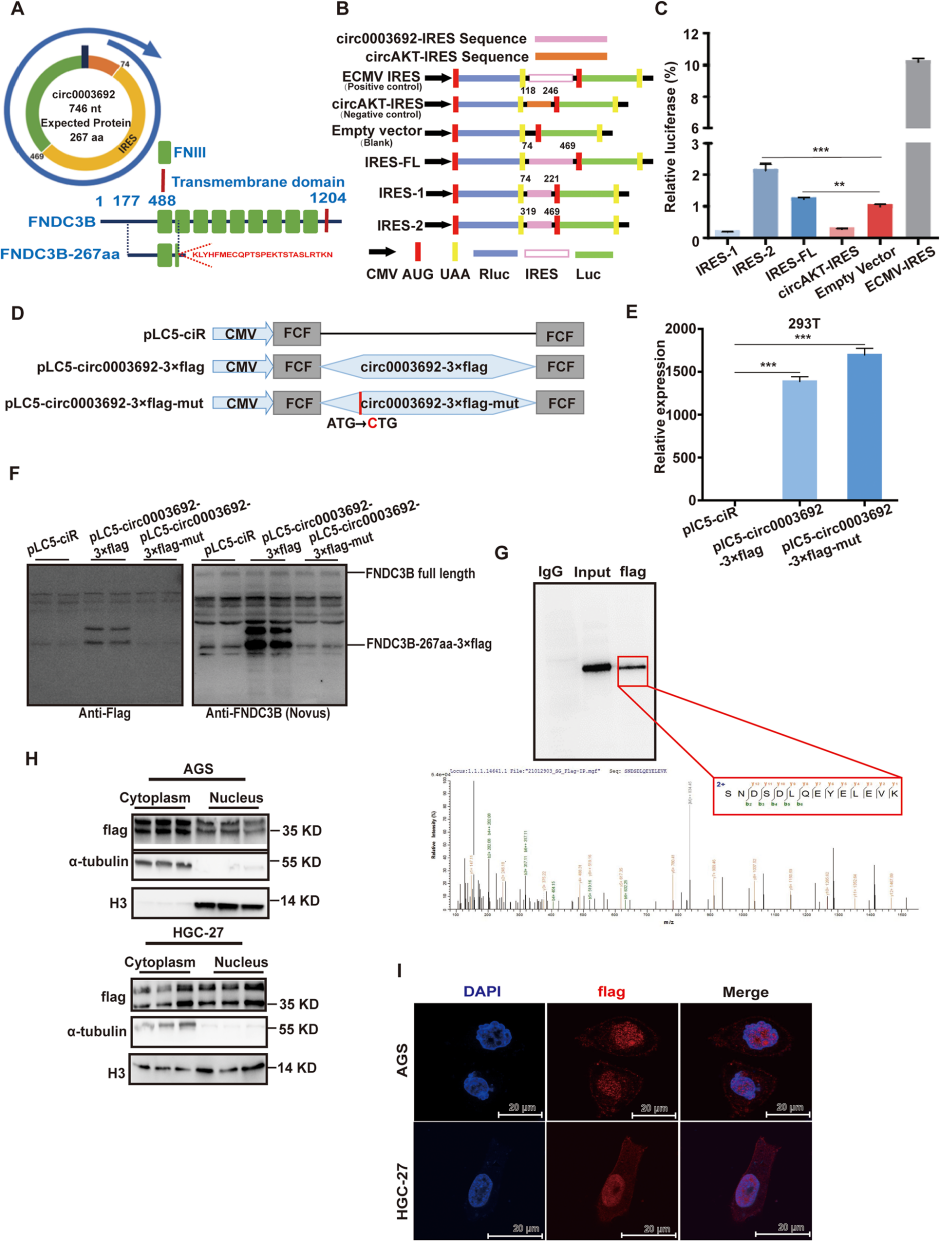
Circ0003692 encodes a novel protein FNDC3B-267aa. **(A)** The predicted 746-nt circ0003692 has the potential to encode a 276-amino acid protein known as FNDC3B-267aa. **(B)** IRES constructs for reporter assay. Full-length IRES, IRES-1, IRES-2, and positive/negative IRES were inserted between the Rluc and hLuc reporters, each equipped with independent start and stop codons. **(C)** Relative luciferase activity was determined through the dual-luciferase reporter assay. **(D)** Construction of circ0003692 expression vector. To establish a detectable circ0003692 expression vector, the flag-tagged circ0003692 sequence was cloned into a CMV-induced expression vector. For the FNDC3B-267aa deleted circ0003692 expression vector, the flag-labeled circ0003692 sequence with start codon mutation (ATG/CTG) was cloned into the CMV-induced expression vector. **(E)** qRT-PCR analysis was conducted to confirm the RNA expression of pLC5-circ0003692-3×flag and pLC5-circ0003692-3×flag-mut vectors. **(F)** Western blotting analysis was conducted to confirm the overexpression of pLC5-circ0003692-3×flag and pLC5-circ0003692-3×flag-mut vectors. **(G)** LC-MS/MS identification of protein FNDC3B-267aa. **(H)** The expression level of FNDC3B-267aa in nuclear and cytoplasmic components of AGS and HGC-27 cells was determined. **(I)** Immunofluorescence was used to determine the localization of FNDC3B-267aa in AGS and HGC-27 cells. **P < 0.01, ***P < 0.001; ns, no significance

The prediction results revealed that circ0003692 might encode a conserved 267 amino acid peptide (Fig. 3A). To validate the predicted IRES activity in circ0003692, we designed a series of two dual luciferase reporter vectors, as depicted in Fig. 3B. The luciferase assay showed full-length IRES and IRES2 significantly induced increased Luc/Rluc activity compared with empty vector. In contrast, IRES1 or the negative vector failed to induce Luc activation, suggesting that IRES2 might be responsible for 5’-cap-independent translation of FNDC3B-267aa (Fig. 3C).

Next, to validate the presence of FNDC3B-267aa, we constructed two vectors: pLC5-circ0003692-3×flag for overexpression, in which circ0003692 ORF was fused with 3×flag-tag; pLC5-circ0003692-3×flag-mut for mutation, in which the predicted start codon “ATG” was mutated to “CTG“(Fig. 3D). qRT-PCR results indicated successful overexpression of circ0003692 when transfecting both circ0003692-3×flag and mut constructs (Fig. 3E). However, western blot analysis showed that FNDC3B-267aa-flag was induced in pLC5-circ0003692-3×flag group, but not in the mut group (Fig. 3F). Further validation of FNDC3B-267aa originating from circ0003692 was performed through IP-MS. This analysis confirmed the presence of FNDC3B-267aa at the predicted molecular weight (35 KD) (Fig. 3G). Moreover, FNDC3B-267aa was primarily localized to the nuclei of GC cells (Fig. 3H-I). Taken together, these results revealed that circ0003692 encoded a novel protein, FNDC3B-267aa.

### Circ0003692 inhibits GC migration in vitro and in vivo by translating FNDC3B-267aa

**Fig. 4:**
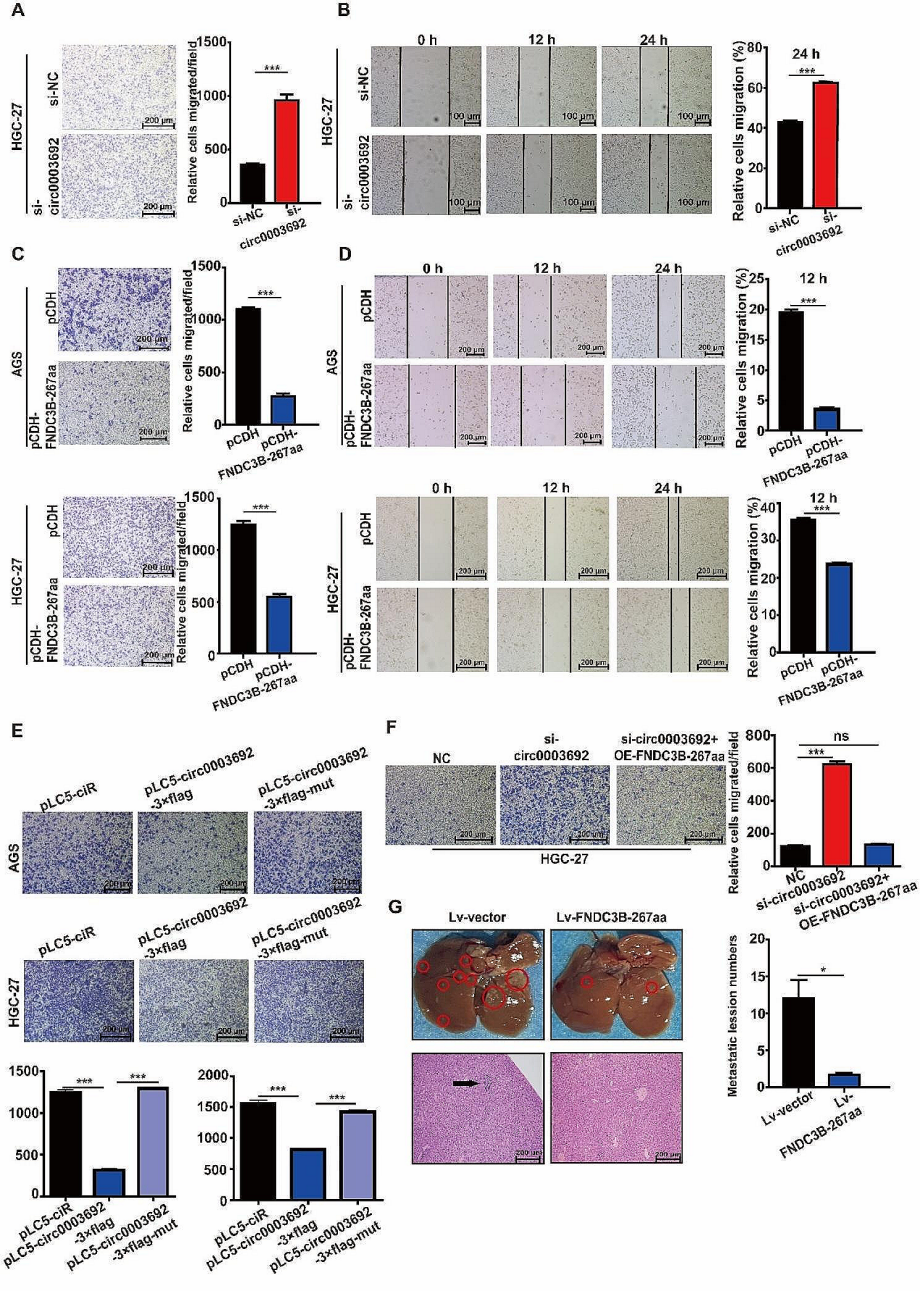
Circ0003692 suppresses GC migration via FNDC3B-267aa in vitro and in vivo. **A-D.** Transwell and wound healing assay were performed to detect the migration ability of GC cells transfected with si-circ0003692 (A-B) and pCDH-FNDC3B-267aa vector (C-D). **E.** Transwell assay were performed to detect the migration ability of pLC5-circ0003692-3×flag and pLC5-circ0003692-3×flag-mut vectors. F. Rescue experiment was used to detect the migration ability of GC cells overexpressing FNDC3B-267aa after interfering with circ0003692. **G.** Representative photographs of the whole liver tissues and hematoxylin-eosin (HE) staining of liver metastatic nodules. Bars, 200 µM. *P < 0.05, ***P < 0.001; ns, no significance

To investigate the biological behavior of circ0003692 in GC cells, siRNAs targeting its junction sites were designed. The expression of circ0003692 were compared across various GC cell line, revealing a relatively high level of circ0003692 in HGC-27 cells (Additional file 2: Fig. S1). Subsequent observation revealed that si-circ0003692 led to no significant difference in FNDC3B mRNA expression (Additional file 2: Fig. S2). Transwell and wound healing assays indicated an enhanced migration ability of HGC-27 cells upon circ0003692 suppression (Fig. 4A-B).

Subsequently, we validated the protein expression of FNDC3B-267aa in various cell line, selecting AGS and HGC-27 cells for subsequent overexpression assays (Additional file 2: Fig. S3). The efficiency of FNDC3B-267aa overexpression was confirmed via western blot analysis (Additional file 2: Fig. S4). Transfection with pCDH-FNDC3B-267aa vector significantly inhibited the migration ability of GC cells, as depicted in Fig. 3C-D. To further confirm the inhibitory effect of circ0003692 on GC cell migration through FNDC3B-267aa translation, we compared the effect of pLC5-circ0003692-3×flag and pLC5-circ0003692-3×flag-mut on cell migration. As shown in Fig. 4E, the absence of a migration inhibition effect was observed in GC cells transfected with the flag ATG-mut vector. Furthermore, a rescue experiment demonstrated that overexpression of FNDC3B-267aa following circ0003692 interference counteracted the increased cell migration induced by si-circ0003692, indicating that circ0003692 inhibited GC cell migration through FNDC3B-267aa protein translation (Fig. 4F). Additionally, it was confirmed that circ0003692 had no effect on the proliferation ability of GC cells (Additional file 2: Fig. S5).

To investigate the metastasis-suppressing effect of FNDC3B-267aa in vivo, we developed a lentivirus overexpressing FNDC3B-267aa to infect GC cells. The overexpression of FNDC3B-267aa and its inhibitory effect on migration were confirmed in the stably transformed GC cell lines (Additional file 2: Fig. S6). To further evaluate the anti-carcinogenic role of circ0003692 in vivo, 6 weeks old BABL/c nude mice were employed to construct a model of in vivo liver metastasis model of human GC. Nude mice were injected with lentivirus overexpressing FNDC3B-267aa AGS cells into their spleens. FNDC3B-267aa overexpression resulted in decreased metastatic lesions in the liver, as indicated by H&E staning (Fig. 4G). In summary, these findings demonstrate that the novel protein FNDC3B-267aa, rather than circ0003692, is responsible for inhibiting the migration of GC cells both in vitro and in vivo.

### FNDC3B-267aa directly interacts with c-Myc protein in the nucleus

**Fig. 5:**
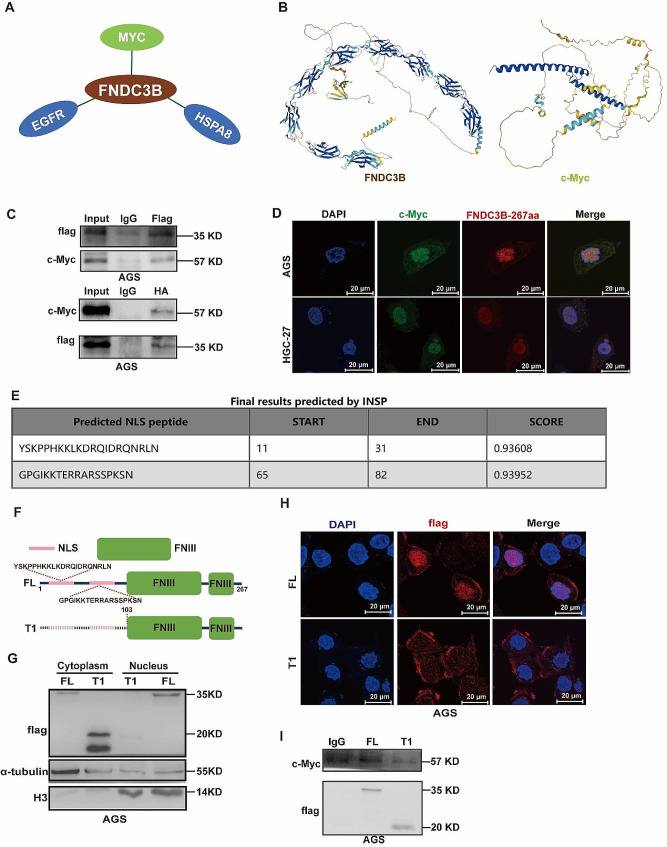
FNDC3B-267aa directly interacts with c-Myc protein in the nucleus. **(A)** An illustration of the protein interaction network involving FNDC3B. **(B)** Representation of the tertiary structure of FNDC3B and c-Myc proteins. **(C)** Confirmation of the interaction between FNDC3B-267aa and c-Myc through Co-IP. **(D)** Verification of the subcellular localization of FNDC3B-267aa and c-Myc through immunofluorescence. **(E)** NLS of FNDC3B-267aa predicted by INJP database. **(F)** Schematic illustrations of FNDC3B-267aa full-length (FL) and truncated mutant (T1) expression vectors. (G) Detection of FL and T1 in nuclear and cytoplasmic of AGS Cells. (H) Localization of FL and T1 via immunofluorescence in AGS Cells. (I) Comparation of the interaction between FL or T1 with c-Myc was detected by Co-IP assay

Given the homology and high structural similarity between FNDC3B-267aa and FNDC3B, we employed the BioGRID database to predict proteins interaction with FNDC3B, resulting in the identification of 187 candidates (Additional file 1: Table S8). As shown in Fig. 5A and Additional file 2: Fig. S7A, we identified MYC, EGFR, and HSPA8 as the proteins most closely associated with FNDC3B. We first validated the combination of FNDC3B-267aa and c-Myc through co-IP experiments. The results indicated that FNDC3B-267aa had a protein interaction with c-Myc (Fig. 5C). Importantly, analysis of the TCGA database revealed upregulation of c-Myc expression in GC (Additional file 2: Fig. S7B), a finding included in Fig. 5A and Additional File 1: Table S8. Additionally, we confirmed that inhibiting c-Myc suppressed the migration of GC cells (Additional file 2: Fig. S7C-D). Furthermore, we validated the interaction between FNDC3B-267aa and c-Myc through Co-IP experiments with anti-flag and anti-HA antibodies (Fig. 5C, Additional file 2: Fig. S8). The immunofluorescence experiment demonstrated that FNDC3B-267aa and c-Myc were co-localized primarily in the nucleus (Fig. 5D). Furthermore, we conducted a preliminary exploration of the mechanism through which FNDC3B-267aa enters the nucleus. Predictions from the INSP database suggested the presence of two nuclear localization sequences (NLS) in FNDC3B-267aa at amino acid positions 11–31 and 65–82 (Fig. 5E). Accordingly, we designed two recombinant proteins: FL containing FNDC3B-267aa-3×Flag and the NLS truncated mutant T1 (Fig. 5F). Subsequently, we confirmed that T1 was mainly located in the cytoplasm, while FL remained in the nucleus (Fig. 5G-H). Furthermore, Co-IP experiment further supported that the interaction between FNDC3B-267aa and c-Myc was obviously inhibited upon NLS deletion (Fig. 5I). Thus, the data above suggested that FNDC3B-267aa could interact with c-Myc, and this binding might require NLS sequence of FNDC3B-267aa.

### FNDC3B-267aa facilitates the proteasomal degradation of c-Myc and consequently suppresses the c-Myc-Snail/Slug axis

**Fig. 6:**
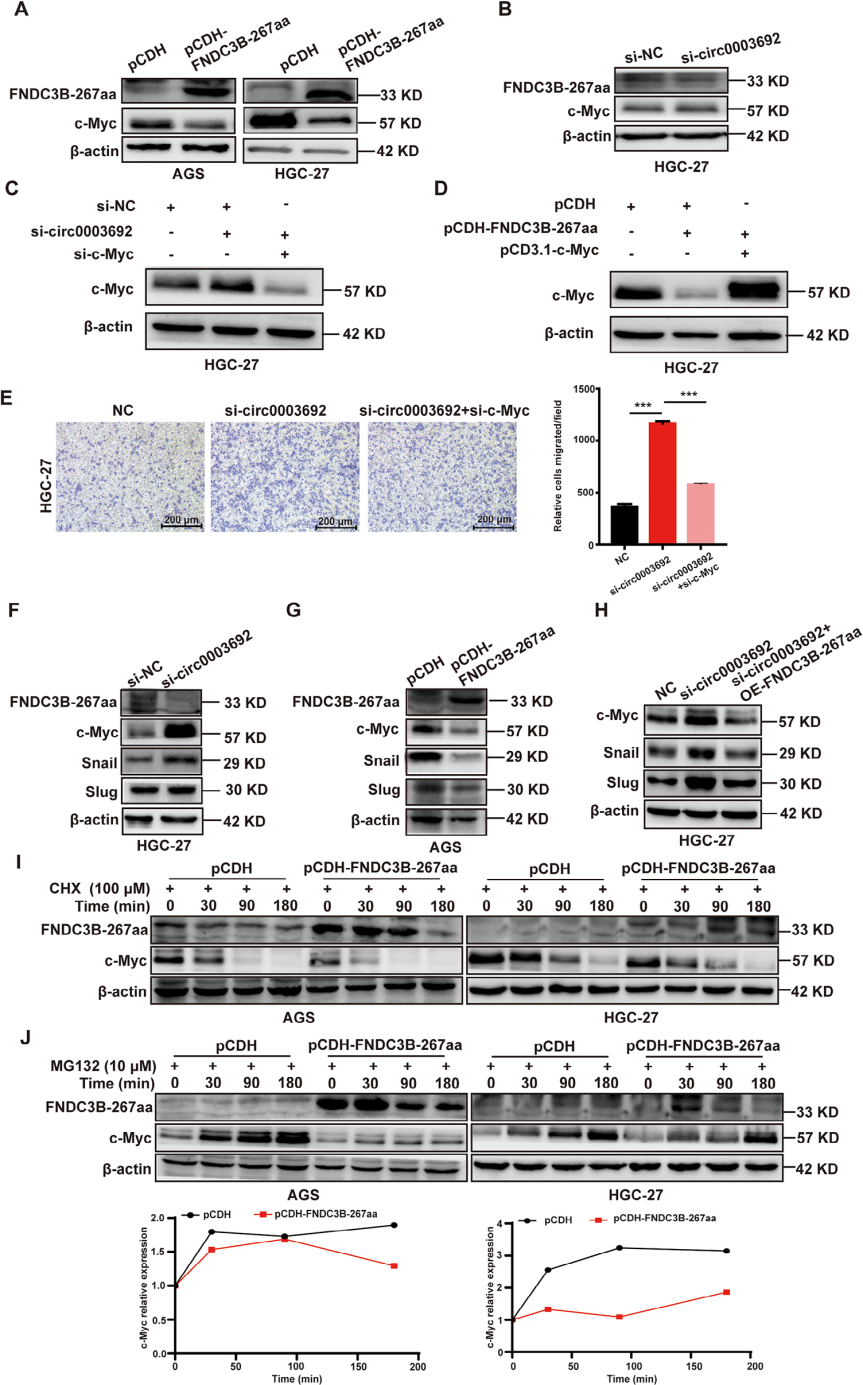
FNDC3B-267aa promotes the proteasomal degradation of c-Myc. **(A)** The influence of FNDC3B-267aa overexpression on c-Myc protein level in AGS and HGC-27 cells. **(B)** The influence of si-circ0003692 on c-Myc protein level in HGC-27. **(C)** The protein level of c-Myc in HGC-27 transfected with si-circ0003692 or si-circ0003692 + si-c-Myc. (D) The protein level of c-Myc in HGC-27 with FNDC3B-267aa overexpression or FNDC3B-267aa + c-Myc overexpression. **(E)** Rescue experiment was used to detect the migration ability of HGC-27 cells interfering with si-circ0003692 or si-circ0003692 + si-c-Myc. **F-H.** Western blot assay was used to validate the effect of FNDC3B-267aa on c-Myc-Snail/Slug axis. **I.** Impact of FNDC3B-267aa on stability of c-Myc protein by using CHX (100 µM). **J.** MG132 rescue experiment was used to confirm the proteasomal degradation of c-Myc by FNDC3B-267aa. ***P < 0.001

Subsequently, we observed that overexpression of FNDC3B-267aa decreased, whereas si-circ0003692 increased the protein level of c-Myc (Fig. 6A-B). Additionally, rescue experiments demonstrated that c-Myc knockdown reversed the up-regulation of c-Myc protein induced by circ0003692 knockdown (Fig. 6C), while c-Myc overexpression reversed the down-regulation of c-Myc protein induced by FNDC3B-267aa overexpression (Fig. 6D). Next, we investigated whether circ0003692-c-Myc axis is involved in the regulation of GC cells migration. We confirmed that simultaneously interfering with circ0003692 and c-Myc impaired the enhancement of GC migration induced by si-circ0003692 (Fig. 6E). These results suggested that the circ0003692-c-Myc axis might be crucial for inhibiting the metastatic function of GC cells. Building upon the previous findings demonstrating c-Myc’s regulation of the EMT pathway through Snail and Slug in GC [18], we additionally verified that FNDC3B-267aa inhibited the expression of Slug and Snail, key downstream targets of c-Myc involved in EMT pathway (Fig. 6F-H).

Interestingly, overexpression of FNDC3B-267aa did not significantly alter c-Myc mRNA levels as detected by qRT-PCR, implying that FNDC3B-267aa regulated c-Myc primarily at the protein level rather than the RNA level (Additional file 2: Fig. S9A). Consequently, AGS and HGC-27 cells were treaed with CHX along with FNDC3B-267aa overexpression to assess the protein stability of c-Myc. A notable decrease in the stability of c-Myc protein was observed following FNDC3B-267aa overexpression in both cell lines (Fig. 6I). This implicated that FNDC3B-267aa might be involved in c-Myc protein degradation in GC. Cellular protein degradation primarily involves two mechanisms: autophagy-lysosome and ubiquitin-proteasome pathways. To further investigate how FNDC3B-267aa influences the degradation pathway of c-Myc protein, we initially confirmed that there was no significant change in the autophagy markers LC3A and LC3B after FNDC3B-267aa overexpression, suggesting that FNDC3B-267aa might not modulate c-Myc through the autophagy degradation pathway (Additional file 2: Fig. S9B). Previous studies have highlighted the importance of the ubiquitination-proteasome pathway in c-Myc protein degradation [22]. Consequently, we explored whether FNDC3B-267aa affects the level of c-Myc protein via ubiquitination-proteasome pathway. It was observed that the proteasome inhibitor MG132 partially reversed the decrease in c-Myc protein induced by FNDC3B-267aa overexpression in GC cells (Fig. 6J). These findings strongly indicated that FNDC3B-267aa regulated the protein expression of c-Myc by promoting its degradation through the proteasome pathway. Furthermore, Co-IP experiment was conducted to compare the ubiquitin modification of c-Myc between pCDH and pCDH-FNDC3B-267aa. Surprisingly, co-IP experiment revealed diminished ubiquitination of c-Myc following FNDC3B-267aa overexpression (Additional file 2: Fig. S9C). In conclusion, these results suggested that FNDC3B-267aa facilitated the proteasomal degradation of c-Myc and consequently suppressed the c-Myc-Snail/Slug axis.

## Discussion

High-throughput sequencing technology and bioinformatics have led to the discovery of numerous new circRNAs in GC cells, some exhibiting differential expression between GC tissues and adjacent normal tissues [19]. For instance, Sui et al. identified 467 differentially expressed circRNAs, comprising 214 significantly upregulated and 253 downregulated circRNAs in GC tissues, using microarray data obtained from 8 GC tissues and adjacent tissues [20]. Dang et al. discovered 306 differentially expressed circRNAs in GC compared to adjacent normal tissues, with 191 upregulated and 522 downregulated circRNAs [21]. Most studies suggested a higher prevalence of downregulated circRNAs in GC tissues compared to upregulated ones [19]. Furthermore, accumulating evidence supported the crucial involvement of circRNAs in the proliferation, apoptosis, migration, invasion, and drug resistance of GC cells [9–11, 22]. Recent studies have revealed that FNDC3B plays a crucial role in various cancers, including acute myeloid leukemia, hepatocellular carcinoma, cervical cancer, and colorectal cancer [23]− [24]. Multiple reports now confirm that FNDC3B can produce different circRNAs during the cleavage process. CircFNDC3B regulates biological functions such as tumor proliferation and metastasis [25]. For example, hsa_circ_0001361 has been reported to accelerate the formation and metastasis of the vascular system in oral squamous cell carcinoma [26], while hsa_circ_0006156 inhibited colorectal cancer stemness and metastasis via RNF41-dependent ASB6 degradation [27]. In current research, we identified a novel circRNA originating from FNDC3B: circ0003692. Circ0003692 was significantly underexpressed in GC and associated with T, N, M grade. Furthermore, we confirmed that circ0003692 inhibited GC migration in vitro and in vivo. Therefore, investigating the function of circRNAs formed by reverse splicing of FNDC3B in GC is crucial.

CircRNA-translated proteins modulate various physiopathologic processes including hepatocellular carcinoma, colon cancers, gastric cancer, and etc. For example, circβ-catenin encodes a 370-amino-acid peptide (β-catenin-370aa), promoting the proliferation and metastasis of liver cancer [28]. CircFBXW7 encodes a 185-amino-acid peptide (FBXW7-185aa), which inhibits the occurrence and development of gliomas [29]. Zheng et al. identified a novel circRNA, circPPP1R12A, which encodes a 73-amino-acid peptide (circPPP1R12A-73aa) and promotes the proliferation and metastasis of colon cancer [30]. Xia et al. recently discovered that circAKT3 encoded a novel protein of 174 amino acids (AKT3-174aa), which interacted with phosphorylated PDK1 and down-regulated the PI3K/AKT signaling pathway [31]. These studies suggest that circRNAs regulate tumor proliferation and metastasis by serving as templates for protein translation. Mounting evidence has revealed abnormal circRNA expression in GC tissues, suggesting its potential application in the diagnosis and treatment of GC [32]. While current researches on the mechanism of GC circRNA primarily focus on the microRNA sponge and RBP binding effects, the translation potential of circRNA in GC has garnered more attention in recent years. For instance, Zhang et al. demonstrated that circDIDO1 encoded a protein of 529 amino acids, which promoted GC cell invasion, migration and proliferation [33]. Similarly, MAPK1-109aa encoded by circMAPK1 inhibited the invasion and proliferation of GC [34]. These findings emphasize the critical role of circRNA-translating proteins in the occurrence and development of GC. In our study, we identified that circ0003692 encoded the FNDC3B-267aa protein and inhibit GC migration through its translation protein FNDC3B-267aa. Similarly, hsa_circ_0006156 from FNDC3B has been reported to inhibit Snail expression by encoding a 218aa protein, thus promoting the tumor inhibitory effect of FBP1 in colon cancer [35].

Identification of the protein interacting with FNDC3B-267aa is crucial due to its role mediated by circ0003692. Specifically, our investigations revealed that FNDC3B-267aa engaged in biologically significant interactions with c-Myc and downregulated c-Myc protein expression through proteasome degradation pathway. The MYC oncoprotein, also known as c-Myc, plays a significant role as a transcription factor in regulating the expression of numerous genes associated with cell proliferation, growth, and metabolic pathways [36]- [37]. C-Myc has a short half-life of only 30 min, after which it undergoes degradation through the ubiquitin-proteasome pathway [38]. However, our research found that FNDC3B-267aa had no influence in the ubiquitin-dependent degradation of c-Myc. In recent years, numerous studies have demonstrated that, in addition to the ubiquitin-proteasome degradation system, certain eukaryotic proteins can degrade via the proteasome in a ubiquitin-independent manner. This process is termed ubiquitin-independent proteasomal degradation (UblnPD). Makaros et al. identified numerous sequences that promoted UbInPD and 69 full-length proteins affected by this process [39]. Wei et al. discovered that RNA-edited AZIN1 retarded c-Myc degradation via the non-ubiquitin-dependent proteasome pathway mediated by OAZ2 [40]. Gu et al. uncovered a novel universal mechanism for the selective degradation of nuclear proteins, circumventing the classical ubiquitination system, termed the midnolin-proteasome pathway [41]. Shi et al. identified that tumor suppressor circPABPC1 physically connected ITGB1 to the proteasome, facilitating non-ubiquitin-dependent degradation of ITGB1 [42]. Hence, further investigation is warranted to determine whether FNDC3B-267aa promotes c-Myc degradation via the UblnPD pathway.

In current study, we confirmed the predominant nuclear localization of FNDC3B-267aa and c-Myc. Since the translation of FNDC3B-267aa via IRES primarily occurs in the cytoplasm, we investigated how FNDC3B-267aa gains access to the nucleus. NLS is a protein domain facilitating protein transport into the nucleus [43]. Using the NLSdb databese, we predicted the presence of two NLS in the FNDC3B-267aa structure with nuclear localization scores of 0.93608 and 0.93952, respectively. Additionally, we predicted three NLS of the nucleoprotein c-Myc on this website with scores of 0.93169, 0.93843, and 0.95384. The NLS score for c-Myc aligns with the previously established understanding of c-Myc’s nuclear localization. Our study confirmed that T1 truncation lacking of two NLS sequences was mainly cytoplasmic, supporting the significant inhibition of the FNDC3B-267aa and c-Myc interaction upon deletion of two NLS sequences. However, we did not independently construct two distinct NLS truncations. Further investigation should focus on identifying accurate amino acid sites related to nuclear cytoplasmic transport for FNDC3B-267aa.

## Conclusion

We screened and identified a novel circFNDC3B, circ0003692, which encoded FNDC3B-267aa protein. Circ0003692 was underexpressed in GC and associated with T, N, M grade. Functionally, circ0003692 inhibited GC migration in vitro and in vivo by translating FNDC3B-267aa. Mechanistically, FNDC3B-267aa interacted with c-Myc and promoted the proteasomal degradation of c-Myc, thereby inhibiting the expression of Snail and Slug. This study suggested that circ0003692 and FNDC3B-267aa could serve as novel therapeutic targets for GC.

### Electronic supplementary material

Below is the link to the electronic supplementary material.

Additional file 1

Additional file 2

Additional file 3

## Data Availability

All data used in this work can be acquired from the corresponding author upon reasonable request.

## Abbreviations

CDS: Coding sequence
circRNA: Circular RNA
DAPI: 4', 6-diamidine-2-phenylindole
DNA: Deoxyribo nucleic acid
GC: Gastric cancer
h: Hour
IF: Immunofluorescence
FNDC3B: Fibronectin Type III Domain Containing 3B
c-Myc: MYC proto-Oncogene, BHLH transcription factor
ISH: In situ hybridization
min: Minute
miRNA: MicroRNA
mL: Milliliter
mRNA: Messenger RNA
NC: Normal control
ncRNA: Non-coding RNA
IRES: Internal ribosome entry site
PCR: Polymerase chain reaction
RBP: RNA binding protein
RNA-FISH: RNA-fluorescence in situ hybridization
RNase R: Ribonuclease R
s: Second
siRNA: Small interfering RNA
TCGA: The Cancer Genome Atlas
TMA: Tissue MicroArray
Tris: Trimethylol aminomethane
μL: Microliter
μM: Micromole
