# Supplementary material for: A novel protein FNDC3B-267aa encoded by circ0003692 inhibits gastric cancer metastasis via promoting proteasomal degradation of c-Myc
Source: J Transl Med. 2024 May 27;22:507. doi: 10.1186/s12967-024-05225-4 (PMC11129431; doi:10.1186/s12967-024-05225-4)
Supplement: Supplementary file 2 — Additional file 2 [file 12967_2024_5225_MOESM2_ESM.docx]

**
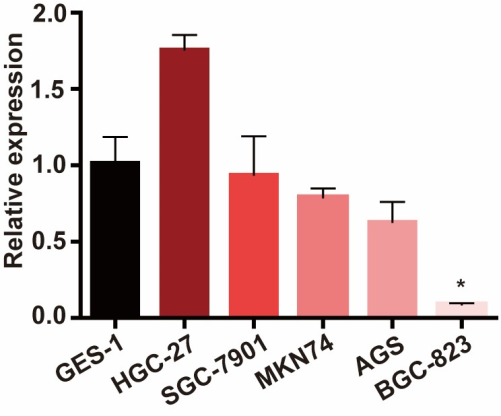
**

**Fig. S1** RNA expression of circ0003692 in GC cell lines. qRT‐PCR was used to verify RNA expression of circ0003692.


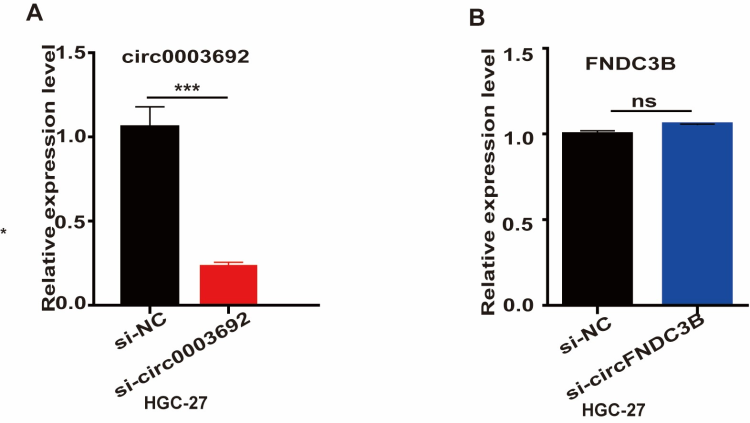


**Fig. S2** Interference efficiency of knocking down circ0003692. **A.** Interference efficiency of si-cicFNDC3B. **B.** The effect of si-cicFNDC3B on FNDC3B mRNA.


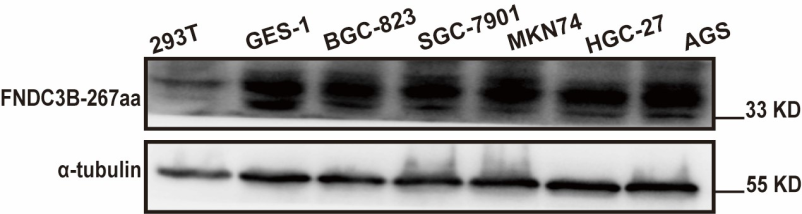


**Fig. S3** Protein level of FNDC3B-267aa in cell lines. Western blot assay was used to verify protein level of FNDC3B-267aa.


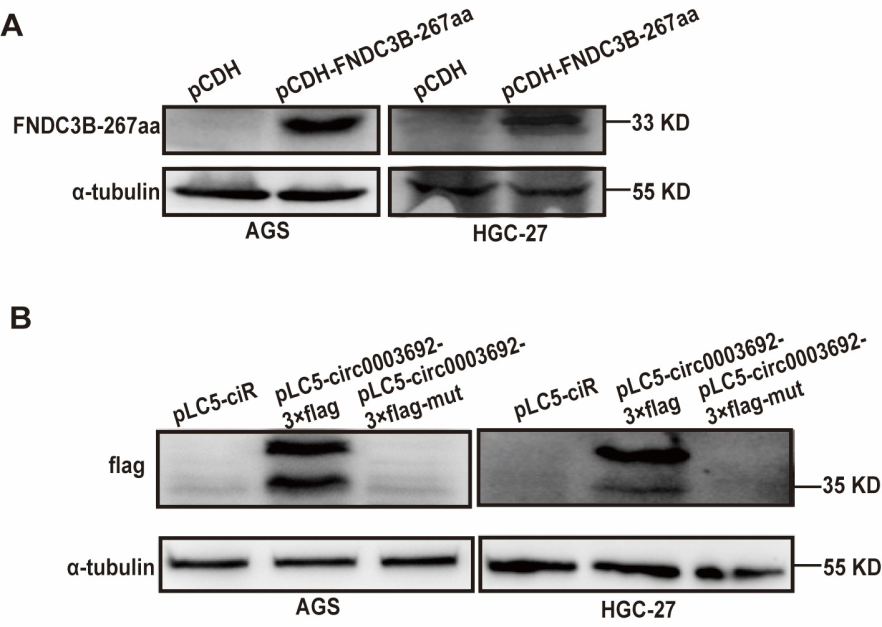


**Fig. S4** The efficiency of FNDC3B-267aa overexpression in AGS and HGC-27 cells by western blot. **A.** Western blot assay was used to detect FNDC3B-267aa overexpression of pCDH-FNDC3B-267aa vector. **B.** Western blot assay was used to detect 3×flag tagged FNDC3B-267aa overexpression of pLC5-circ0003692-3×flag and pLC5-circ0003692-3×flag-mut vectors.


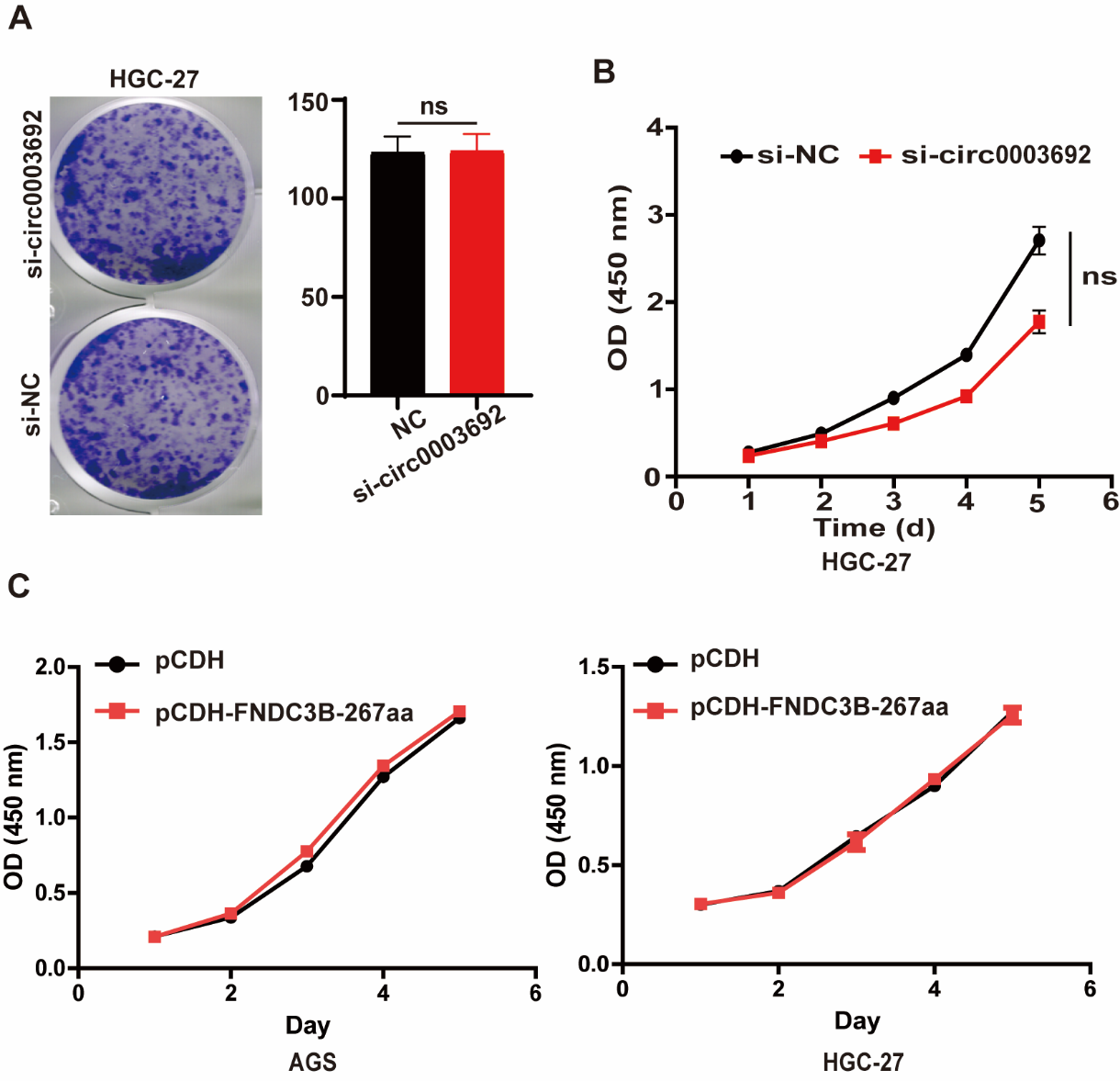


**Fig. S5** Effect of circ0003692 on the proliferation ability of GC cells. **A-C.** The effect of circ0003692 on proliferation was detected by plate cloning and CCK8 experiments by knocking down circ0003692 and overexpressing FNDC3B-267aa.


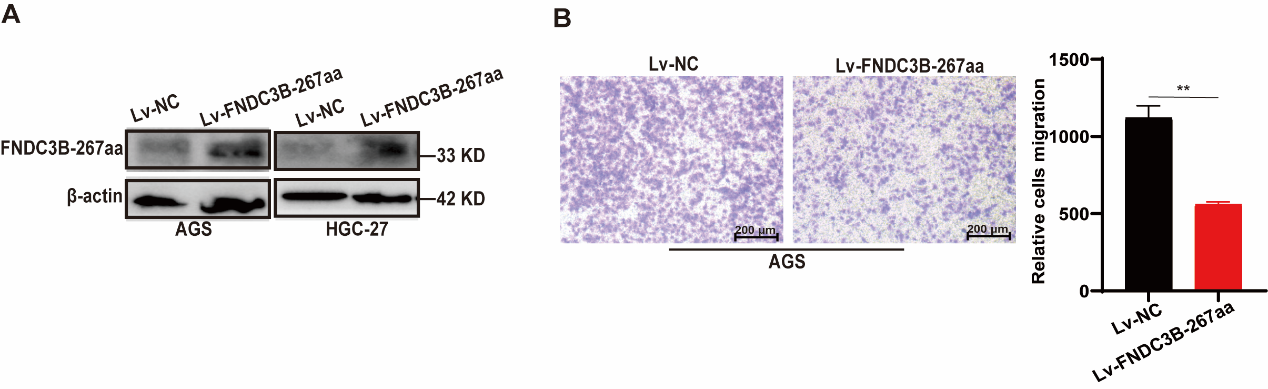


**Fig. S6** The overexpression of FNDC3B-267aa and the inhibiting-migration effect of the stably transformed AGS and HGC-27 cell lines. **A.** FNDC3B-267aa overexpression of stably transformed AGS and HGC-27 cell lines was detected by western blot assay. **B.** The effect of stable transfection of FNDC3B-267aa on the migration of AGS cells was verified by transwell experiment.


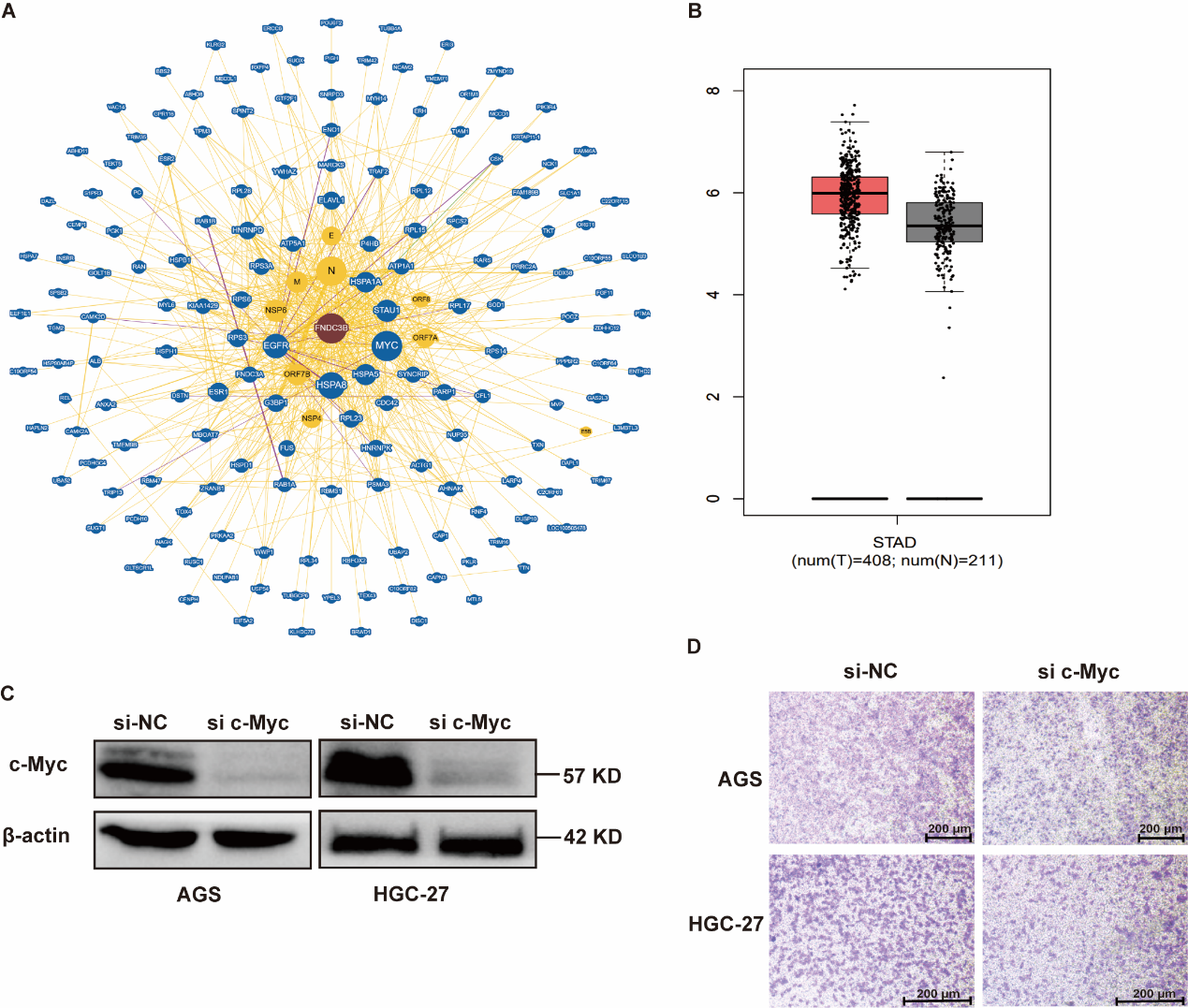


**Fig. S7** Expression and biological function of c-Myc in GC. **A.** Interaction network of FNDC3B. The yellow circle represents genes from different species, while the blue circle represents genes from the same species. A larger circle indicates a higher likelihood of interaction. **B.** c-Myc expression were verified by TCGA database. **C.** The effect of si-c-Myc was tested by western blot assay. **D.** The effect of si-c-Myc on the migration in GC cells was verified by transwell experiment.


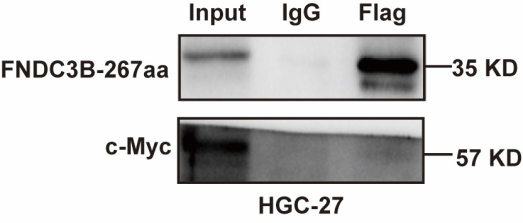


**Fig. S8** Interaction between FNDC3B-267aa and c-Myc in HGC-27. Western blot assays identified the efficiency of Co-IP assay.


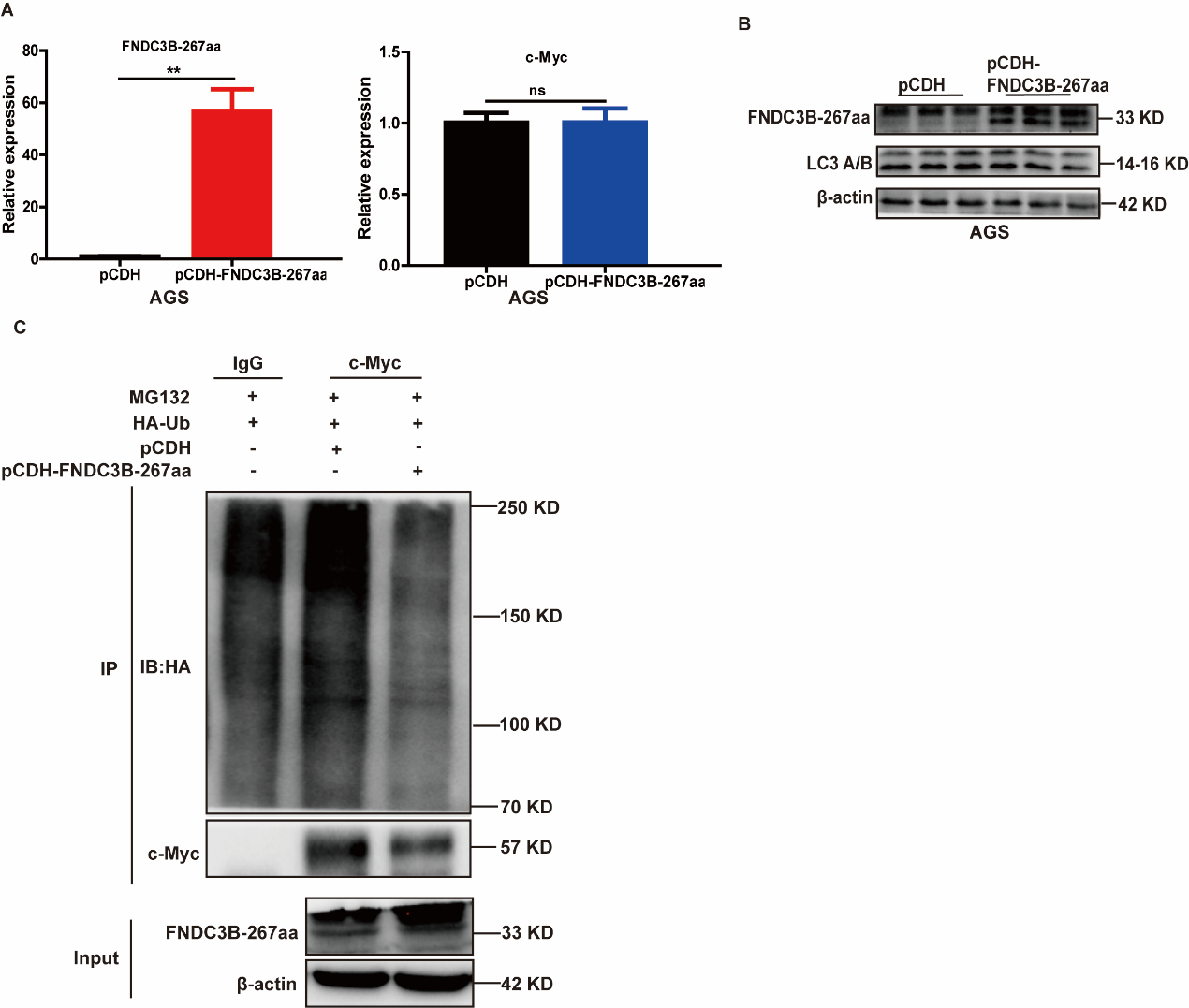


**Fig. S9** Effect of FNDC3B-267aa on c-Myc mRNA (A), LC3 A/B protein level (B), and c-Myc ubiquitination level (C).
