# Supplementary material for: A novel protein FNDC3B-267aa encoded by circ0003692 inhibits gastric cancer metastasis via promoting proteasomal degradation of c-Myc
Source: J Transl Med. 2024 May 27;22:507. doi: 10.1186/s12967-024-05225-4 (PMC11129431; doi:10.1186/s12967-024-05225-4)
Supplement: Supplementary file 3 — Additional file 3 [file 12967_2024_5225_MOESM3_ESM.docx]

**Supporting materials and methods**

**1. Cell lines and cell culture**

All GC cell lines MKN74, HGC-27, AGS, along with normal human embryonic kidney cells 293T and human gastric mucosal epithelial cells GES-1 were obtained from the Chinese Academy of Sciences (Shanghai, China). 293T, GES-1 and MKN74 were cultured in DMEM/high glucose, AGS in DMEM/F12, HGC-27 in RPMI-1640 medium. All cell lines were supplemented with 10% FBS, under standard culture conditions (5% CO_2_, 37 °C).

**2. Actinomycin D assay**

The stability of circ0003692 and linear FNDC3B mRNA was compared by actinomycin D assay. 293T cells were treated with actinomycin D (5 μg/mL) (Genview, Beijing, China) for durations of 0,3,6, and 9 hours. Then, cells were collected and lysed using RNAiso Plus (Takara, Kyoto, Japan), followed by RNA extraction and qRT-PCR analysis.

**3. RNase R treatment**

AGS and HGC-27 cells in good condition were collected. Subsequently, total cell RNA was extracted using RNAiso Plus and incubated at 6°C with or without RNase R (6 U) (Lucigen, Middleton, WI, USA) for 37 min, followed by 5 seconds incubation at 85°C. After treatment, the expression level of linear FNDC3B and circ0003692 was evaluated by qRT-PCR.

**4. Cell Counting Kit-8 assay**

After transfection treatment, GC cells (1×10^3^ cells) were seeded in each well of the 96-well plate. Cell viability was measured via the CCK-8 reagent (Topscience, Shanghai, China) every 24 h by measuring the absorbance of different cell lines at 450 nm. The obtained data were counted by Graphpad 7.0.

**5. Flat plate colony formation assay**

After 48 h of treatment, 1×10^3^ GC cells were seeded in 12-well plate to evaluate the proliferation ability of GC cells. After 12 days, GC cells were fixed with 4% paraformaldehyde and then stained with 1% crystal violet. The number of colonies was counted by ImageJ.

**6. Western blotting analysis**

GC cells were lysed with RIPA buffer supplemented with protease inhibitor cocktail and 1% PMSF. Furthermore, cytoplasmic and nuclear proteins were isolated using Nuclear and Cytoplasmic Protein Extraction Kit (Beyotime, Shanghai, China). Samples were separated with 10%, 12%, 15% SDS-PAGE. Protein was transferred to PVDF membranes and incubated overnight at 4°C with primary antibodies, followed by incubation with a secondary antibody at room temperature for 1 h. The antibodies used were as follows: Rabbit anti-FNDC3B/FNDC3B-267aa (Novus, Colorado, USA); Mouse anti-flag (Beyotime, Shanghai, China); Rabbit anti-c-Myc (Abcam, Cambridge, UK); Rabbit anti-USP28 (Abcam, Cambridge, UK); Rabbit anti-Snail (CST, Boston, USA); Rabbit anti-Slug (CST, Boston, USA); Mouse anti-β-actin and anti-α-tubulin (Beyotime, Shanghai, China).

**7. Co‑immunoprecipitation (Co‑IP) assay**

The Pierce Crosslink Magnetic IP/Co-IP Kit (88805, Thermo Fisher scientific) were used for immunoprecipitation and co‑immunoprecipitation. Cells were lysed with lysis buffer and supernatant was collected after centrifugation at 13,000 g for 10 min. 25 µL pierce protein A/G magnetic beads and 100 µL prepared anti-body fluid (5 µg antibody) were added to the centrifuge tube and incubated on a rotary for 15 min at room temperature. DSS cross-linked magnetic bead-bound antibodies were used to add 500 µL of the lysed protein. 500 µL of lysate protein was added to a centrifuge tube containing antibody cross-linked magnetic beads and incubate in a rotary at room temperature at 4℃ overnight. Finally, 100 µL eluent buffer was added to retain the supernatant containing the target antigen. To neutralize the low pH, add 10 µL of neutralization buffer for each 100 µL of eluate, followed by western blotting analysis or mass spectrometry analysis at Sinotech genomics (Shanghai, China). HRP-conjugated Goat Anti-Rabbit IgG Heavy Chain (AS063, Abclonal) was used to avoid the detection of heavy and light chains.

**8. Dual‑luciferase reporter assay**

Sequence of promoters were cloned into the Luc2-IRES-Report vector (Geneseed, Guangzhou). Firefy and Renilla luciferase activity were detected using a Dual-Glo luciferase assay kit (Promega, USA). 24 h after co-transfection, 75 μL of dissolved Dual-Glo Reagent was added for lysis. The fluorescence intensity was detected by the microplate reader, and the fluorescence was achieved by Firefly luminescence. Finally, 75 μL pre-mixed Stop & Glo Reagent was added, and the Renilla luminescence was measured after static for 10 min. A ratio of Firefly luminescence/Renilla luminescence was calculated to determine relative luciferase activity.
